# The Use of Attractants to Stimulate Neonatal Piglet Interest in Rope Enrichment

**DOI:** 10.3390/ani12020211

**Published:** 2022-01-17

**Authors:** Emiline R. Sundman, Nicholas K. Gabler, Suzanne T. Millman, Kenneth J. Stalder, Locke A. Karriker, Anna K. Johnson

**Affiliations:** 1 Department of Animal Sciences, Iowa State University, Ames, IA 50011, USA; esundman@iastate.edu (E.R.S.); ngabler@iastate.edu (N.K.G.); stalder@iastate.edu (K.J.S.); 2 Department of Veterinary Diagnostic & Production Animal Medicine, College of Veterinary Medicine, Iowa State University, Ames, IA 50011, USA; smillman@iastate.edu (S.T.M.); karriker@iastate.edu (L.A.K.); 3 Department of Biomedical Sciences, College of Veterinary Medicine, Iowa State University, Ames, IA 50011, USA; 4 Swine Medicine Education Center, College of Veterinary Medicine, Iowa State University, Ames, IA 50011, USA

**Keywords:** environmental enrichment, piglet, livability, attractants, neonate, preweaning mortality, crushing

## Abstract

**Simple Summary:**

Piglet crushing is one of the leading causes of preweaning mortality. This loss represents reduced production efficiency, substantial economic losses for producers, and is an animal welfare concern. The goal of this study was to determine if enrichment ropes would entice neonatal piglets away from the sow and reduce preweaning mortality. Three treatments (OIL: sunflower oil; MC: milky cheese; SC: semiochemical) were applied to the enrichment ropes to increase attractiveness to piglets. Results indicate that neonatal piglets were interested in all enrichment treatments on Day 2 of life, although there was high individual variation in frequency and duration of interactions. Enrichment treatment did not impact the frequency or duration of rope interactions or litter average weight gain. Piglet mortality was impacted by treatment: MC piglets had the lowest percent mortality during the enrichment period, and SC piglets had the lowest percent mortality over the entire experimental period. This proof-of-concept study highlights the value of neonatal piglet environmental enrichment.

**Abstract:**

In the United States swine industry, preweaning mortality represents the highest mortality rate of any production phase, nearly half attributed to crushing. The overarching aim of this study was to determine if enrichment ropes would entice neonatal piglets away from the sow and reduce preweaning mortality. Rope enrichments were provided to 161 piglets from 26 sows after farrowing. Ropes were dipped in sunflower oil (*n* = 7), semiochemical (*n* = 8), or milky cheese (*n* = 11). Piglet purposeful rope investigations, weight gain, and mortality were recorded. On Day 2, 75% of piglets touched the enrichment at least once, and frequency ranged from 1 to 21 investigations across all treatments. Frequency (*p* = 0.20) and duration (*p* = 0.21) of investigations were not affected by treatment. Preweaning litter average weight gain did not differ between treatments (*p* = 0.71). MC (milky cheese) piglets had the lowest percent mortality when the enrichment ropes were present (Days 2 to 5, *p* = 0.01), and SC (semiochemical) piglets had the lowest percent mortality after the enrichment ropes were removed (Days 6 to weaning, *p* < 0.0001). This proof-of-concept study highlights the potential value of neonatal piglet environmental enrichment.

## 1. Introduction

In the United States (USA) swine industry, preweaning deaths represent the highest mortality rate of any production phase at approximately 17.5% [1]. This reduces production efficiency and incurs substantial economic losses for producers as well as being an animal welfare concern. A 2012 nationwide survey of the USA swine industry reported that nearly half of all preweaning mortality was attributed to piglet crushing [2]. Newborn piglets are more susceptible to crushing due to their small physical size, limited energy reserves, and poor internal temperature regulation [3]. Piglets display cyclic behavior that includes suckling and resting in warm locations [4]. Suckling and heat seeking can be accomplished by resting close to the sow, which in turn can increase crushing risk. Thus, in the first 72 h of a piglet’s life, they are especially vulnerable, and this is when most crushing deaths occur [3].

Previous research to reduce preweaning mortality, specifically crushing, has often focused on altering sow behavior and the farrowing environment. For instance, management practices such as modified pen designs, farrowing assistance, and cross-fostering, as well as additions to the farrowing environment such as neoprene mats and nest building material have been investigated. Sow maternal behavior and genetics as they relate to crushing have also been explored [5]. While some of these methods have shown positive effects on decreasing crushing, implementation of these practices is challenging and piglet crushing remains an issue. 

It has been suggested that if piglets could be enticed away from the sow, their survival rate would increase [6]. Environmental enrichment (EE) is one approach reported in the scientific literature. Environmental enrichment can be defined as “an improvement in the biological functioning of captive animals resulting from modifications to their environment” [7]. Previous work to reduce crushing by enriching the piglet’s environment is more limited than the sow-related literature. The addition of an artificial udder [8], additional heating resources [9] and sensory enrichment such as blue lighting and thyme scent [10] have been successful in attracting piglets but did not reduce preweaning mortality. Thus, a model that can both successfully attract piglets and alter crushing incidence still needs to be identified and validated. 

Weaner and grower pigs are attracted to enrichment materials that are odorous, deformable, destructible, and chewable [11], and hanging objects at eye level have also been shown to maintain pig interest [12]. Pigs display oral-nasal-facial behaviors [13], which means that they will use their mouths and snouts to interact with their environment. Compared to other areas of the body, the pig’s snout has the highest density of tactile mechano-receptors, and they have a very highly developed sense of smell [14]. These traits could be manipulated to draw pigs towards objects through the application of odorous products. For example, weaned pigs preferred feeders sprayed with sow fecal semiochemicals, and their application significantly reduced aggression and tended to increase feeding behavior [15]. A milky cheese attractant was preferred by piglets during lactation after prenatal flavor exposure [16]. Adding flavors, including milky cheese, to creep feed increased preweaning feed intake, post-weaning average daily gain (ADG) and gain-to-feed (G/F) ratio [17]. To our knowledge, neither attractant has been presented through a neonatal piglet enrichment model.

Based on the critical need to improve preweaning livability rates, this proof-of-concept study aimed to determine if enrichment ropes would entice neonatal piglets away from the sow and reduce preweaning mortality. There were three specific objectives to achieve this aim: (1) to describe piglet enrichment use; (2) to evaluate the impact of three attractants on piglet enrichment use; (3) to compare piglet growth rate and livability among the three attractants. 

## 2. Materials and Methods

All experimental procedures were approved by the Iowa State University Animal Care and Use Committee (IACUC-20-054). The research was performed at the Iowa State University Allen E. Christian Swine Teaching Farm, Ames, IA, USA (https://www.ans.iastate.edu/allen-e-christian-swine-teaching-farm (accessed on 14 September 2019).

### 2.1. Animals and Husbandry 

**Figure 1:**
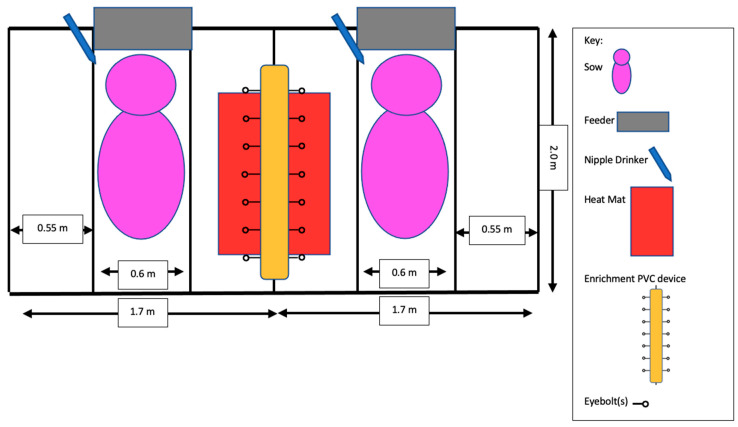
Diagram of two farrowing stalls with one PVC (polyvinyl chloride) device between them. Enrichment ropes were hung off the eyebolts connected to each PVC device.

A total of 161 piglets from 26 sows were used over two replicates. Sows and litters of varying breeds (Commercial Crossbred = 22; Duroc = 4) and parities (average parity 3.9; range 1–11) were used. Each replicate took place over approximately 10 days in July and August 2020. Sows were provided a minimum of a 72 h acclimation to the stall prior to farrowing. Total stall area measured 2.0 m in length × 1.7 m in width with interlocking plastic flooring. The center sow area measured 2.0 m in length × 0.6 m in width with two creep areas measuring 2.0 m in length × 0.6 m in width on either side. A heat mat (Baby Pig Heat Mat-Single 48. MAT; 85 W; 1.2 m in length × 0.3 m in width, polyethylene; Kane Manufacturing, Pleasant Hill, IA, USA) was provided in one creep area, and 1.2 m in length × 0.4 m in width of solid flooring was located under and around the heat mat (Figure 1). Stalls were distributed across two farrowing rooms (seven stalls per room) in a negative pressure, mechanically ventilated barn where the temperature was set at 21.1 °C. Artificial lighting was provided on a night/day schedule, with all lights on during the day (07:00–22:00 h) and only safety lights on from 22:01 to 06:59 h.

Sows were provided ad libitum access to water via one 2 cm nipple and were hand-fed once daily prior to farrowing. Post-farrowing, sows were hand-fed to appetite three times daily in 0.90 kg increments. All diets were prepared by a commercial feed mill (Key Cooperative, Gilbert, IA, USA) composed of primarily corn, soybean meal and dried distillers grains and nutrients formulated to meet or exceed lactating sow nutrient requirements [18]. Farm staff performed sow and litter observations at 07:00 and 15:00 h daily, and at regular intervals during active farrowing. Piglet processing occurred 1 day post-farrowing according to farm standard operating procedures. Piglets were identified by markings on their backs using a livestock-safe paint for identification on video (Prima Tech^®^, Kenansville, NC, USA). Weaning age ranged from 15 to 22 days due to all-in/all-out management procedures. 

### 2.2. Enrichment Device

**Figure 2:**
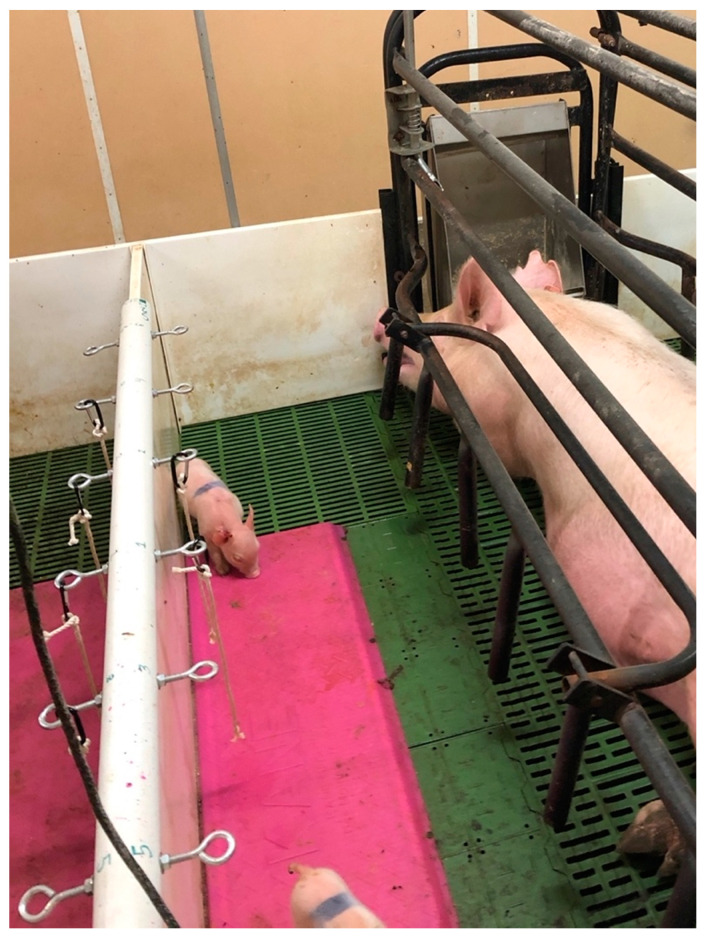
Photograph of the PVC (polyvinyl chloride) device used to hang enrichment ropes. The device, which measured 1.7 m in length, had a 2.7 cm cutout taken out of the bottom of the PVC. This cutout was placed over the top of the 2.5 cm thick plastic siding between two farrowing stalls. In this figure, the eyebolt locations visible (from bottom to top of the photo) are 5, 3, 1, 2, 4, 6.

The enrichment device consisted of a polyvinyl chloride (PVC) pipe from which seven ropes were suspended (Figure 2). To make the enrichment device, a 5 cm in diameter PVC pipe (Plumb Supply Company, Ames, IA, USA) was cut to 1.7 m in length. A 2.7 cm in width slit was created lengthwise down the PVC pipe to allow the pipe to be placed over the 2.5 cm in width plastic farrowing stall divider. Seven eyebolts were attached to each enrichment device spaced 23.0 cm apart for enrichment rope attachment. A 0.8 cm in diameter thread eyebolt (Prime-Line^®^, 9066592 Eye Bolts with nuts, 0.8 cm × 8.3 cm, Redlands, CA, USA) was threaded with a 0.8 cm nut and washer, and then threaded through the pre-drilled holes in the PVC pipe. A second a 0.8 cm nut was threaded onto the end of the eyebolt on the inside of the pipe. The finished PVC pipe was secured to the stall wall using threaded eyebolts and placed over the heat mat. Three stranded, 0.5 cm in diameter plain cotton ropes (R and W Rope, New Bedford, MA, USA) were tied with a modified noose knot to a 5.7 cm in height carabiner hook (OnDepot, Cypress, CA, USA), trimmed to a length of 33.0 cm from the carabiner hook, and the last 2.5 cm of each rope was frayed. The cotton rope is a chewable material, which may suffice to be destructible and deformable and can be presented to the neonatal piglet at eye level. The cotton rope chosen was 0.5 cm in diameter, a size that can fit in the neonatal piglet’s mouth and it was 100% natural and biodegradable. This is an important consideration, as the enrichment must not cause potential or actual harm to the animal or environment. The rope length was also considered. Before project implementation, 2–5-day-old neonatal piglets had their shoulder height measured, and this value was determined to range between 13 and 18 cm. Therefore, the length of the enrichment ropes was designed to hang at a standing neonatal piglet’s shoulder level, or eye level. In addition, the rope end was frayed, and we hypothesized that this may attract the piglet [19]. Distance between hanging ropes was determined based on the width of the 2–5-day-old neonatal piglets’ shoulders. Average shoulder width was 9 cm, and this was doubled so ropes were hung 20 cm apart, allowing two piglets to engage in one rope at the same time.

### 2.3. Treatments

We hypothesized that an absorbent hanging cotton rope dipped in piglet-relevant attractants would be an effective enrichment. Therefore, sows and their litters were assigned to one of three treatments: (1) sunflower oil (OIL, *n* = 7 sows, 41 piglets), (2) semiochemical (SC, *n* = 8 sows, 51 piglets), or (3) milky cheese (MC, *n* = 11 sows, 69 piglets). Treatment assignment was pseudorandom, balancing for sow parity and breed. The sunflower oil was sourced from Blossom Bulk Ingredients (Chicago, IL, USA), and was a mid-oleic, high-linoleic variety. The milky cheese and semiochemical were designed as oil-based mixtures, and sunflower oil was chosen as a relatively neutral, odorless oil. The SC treatment, which contained myristic acid, skatole, and sunflower oil mixed at a set ratio, was adapted from a previous study by Aviles-Rosa et al. [15]. Myristic acid and skatole were sourced from Sigma Aldrich Corp (St. Louis, MI, USA). Once mixed, the SC treatment was refrigerated at 4.5 °C until it was needed on farm. The SC was allowed to come to room temperature before being applied to the enrichment. The MC treatment was sourced from Lucta, LLC (Barcelona, Spain) and mixed into the same sunflower oil as the base treatment. The MC treatment was mixed in three steps. First, the pure MC essence (Luctarom ref. 5788) was mixed with sunflower oil at a ratio by weight of 80% oil to 20% MC essence. Mixing occurred in a thick plastic container, (OXO Good Grips POP Container 3.5 Liter, New York, NY, USA) and the mixed product was stored out of direct light at room temperature. This mixing created the diluted product (Luctarom ref. 5735Z). To create the final MC treatment, 1000 g of sunflower oil was added to a sealable container (Dilabee Round Jug Style Container 2.2 L, Amazon.com (accessed on 4 May 2020), USA), and 0.75 g of the diluted product was added to the same container and swirled gently by hand to combine. Mixing and storage of the milky cheese treatment occurred at room temperature. Treatments were mixed on an as-needed basis throughout the experimental period. 

**Figure 3:**
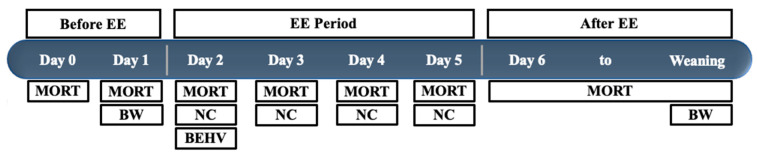
Timeline of the experimental period, and when each measure was collected. Day 0 began when the sow started to farrow, and each additional day begins 24 h after. The environmental enrichment (EE) ropes were placed in the stalls at the beginning of Day 2 and removed at the beginning of Day 6. Methods include BW (piglet body weight collected), BEHV (behavior observations collected), NC (dead piglets necropsied) and MORT (count and percent mortality collected).

Treatment ropes were first applied to the stall at the start of Day 2, replaced with a new rope and fresh treatment at the start of Days 3, 4 and 5, and removed at the beginning of Day 6 (Figure 3). Day 2 was defined as 48 h after the morning or evening the sow began farrowing. If the sow began farrowing in the morning (between 00:00 and 11:59 h), treatment application occurred for that sow each morning (between 08:30 and 09:30 h) on Days 2 to 5. If the sow began farrowing in the evening (between 12:00 and 23:59 h), treatment application occurred for that sow each evening (between 20:30 and 21:30 h) on Days 2 to 5. Treatments were applied by soaking the bottom 20 cm of each rope in the treatment solution for 15 s, and then hanging the rope to air dry for 10 min. Ropes were applied to the stall in one of the seven numbered locations on the enrichment device. Previous work has indicated that piglets suckle and interact with enrichment in a synchronous behavioral pattern, meaning many piglets will engage in the same behavior at the same time [20,21]. Finally, rope to piglet number per litter was standardized over treatments, with one rope per two piglets. This was implemented so that access to the device was standardized, regardless of litter size. Pigs are a hierarchical social species that may monopolize valued resources such as teats and enrichment [22,23,24].

### 2.4. Behavioral Observations 

Color video was recorded continuously (30 frames/s) from Days 2 to 5. Video was recorded using 14 Sony HD Handycam cameras (Model HDR-CX440, San Mateo, CA, USA) mounted on the ceiling so that one camera captured two farrowing stalls with their enrichment devices. Cameras were inspected and reset every 12 h, and secure digital (SD) cards were switched out every 48 h. Video was uploaded to an external data management system (Box.comTM, Redwood City, CA, USA) under Iowa State University privileges. 

The sampling protocol was continuous sampling of all piglets in the litter on Day 2 during the daylight hours (07:00–22:00 h). Frequency and duration of rope investigations, defined as purposeful snout contact with the rope with the mouth open or closed, were recorded. Observers were blinded to all identifying information (sow ID, treatment, room, date, and time of day). Video clips were assigned a random number presented to the observers in a randomized sequence. Videos were assigned to observers in groups of 20, and two video clips were repeated within each set of videos for inter- and intra-observer reliability calculations.

A total of six observers were trained to collect behavioral data. One researcher (ES) with 2 years of behavioral research experience was responsible for training and served as the gold standard when assessing interobserver reliability. Observer reliability was calculated using an index of concordance, as a proportion of all agreements (A) and disagreements (D) in behavioral occurrences between observer and trainer, with the formula (A/(A+D)) × 100 ≥ 85% [25]. Once observers reached ≥85% reliability agreement with the trainer on the reliability test, data collection began using the blinded videos. 

Rope length and end fray length were assessed daily and used as indirect enrichment use measurements. Rope length and end fray length were recorded when placed into the farrowing stall and upon removal using a measuring tape (Stanley Tools, New Britain, CT, USA). 

### 2.5. Mortality and Weight Gain

Piglets were counted at birth and weighed at processing (Day 1) and weaning. Piglet body weight at birth and weaning was used to calculate litter average weight gain. Piglet mortality data were collected daily from birth to the day of weaning and used to calculate total preweaning mortality for comparison to farm and industry standards. Preweaning mortality was defined as piglets that were born alive but died before weaning and calculated as follows: (number dead at weaning / number alive at birth) × 100. To evaluate potential impacts of the enrichment device on piglet mortality, mortality data were further analyzed during three time periods: Days 2 to 5 (during enrichment presence), Days 6 to weaning (after enrichment removal), and Days 2 to weaning (during and after enrichment presence). 

Necropsies were performed on all piglet mortalities that occurred between Days 0 to 5. The stomach was examined to determine if milk was present. External signs of trauma such as bruising, slat marks, blood, and compression of parts of the body were also recorded when present. 

### 2.6. Statistical Analysis 

The litter was considered the experimental unit. All data were checked for normality by plotting a predicted residual plot and a quantile-quantile plot using the PROC UNIVARIATE procedure (SAS Stat. Ver. 9.3, 2011, SAS Institute, Inc., Cary, NC, USA). Data that met the assumption of normality were analyzed using mixed model methods (PROC MIXED). Data with a Poisson distribution were analyzed using generalized linear mixed model methods (PROC GLIMMIX). A *p* ≤ 0.05 was considered significant and PDIFF option was used to separate means when fixed effects were a significant source of variation. Results with *p* ≤ 0.10 were considered statistical trends. 

Frequency and duration of piglet investigations of the enrichment ropes were analyzed using generalized linear mixed model methods (PROC GLIMMIX). Two behavioral models were implemented, one on total frequency and one on total duration. Both models included a fixed effect for treatment (OIL, MC, SC), a linear covariate for litter size on Day 2 (immediately before rope placement), and a random effect for Sow ID. 

Piglet weight gain was analyzed using mixed model methods (PROC MIXED). The statistical model was implemented on the average weight gain of each litter. The model included a fixed effect for treatment (OIL, MC, SC) and linear covariates for average birth weight (kg) and age at weaning (days). 

Preweaning mortality was analyzed using generalized linear mixed model methods (PROC GLIMMIX). Litter percent mortality was analyzed for three time periods: Days 2 to 5, Days 6 to weaning, and Days 2 to weaning. The statistical model included fixed effects for treatment (OIL, MC, SC) and room (1 or 2), and a linear covariate for litter size on Day 2 (immediately before rope placement). 

## 3. Results

### 3.1. Behavior

**Figure 4:**
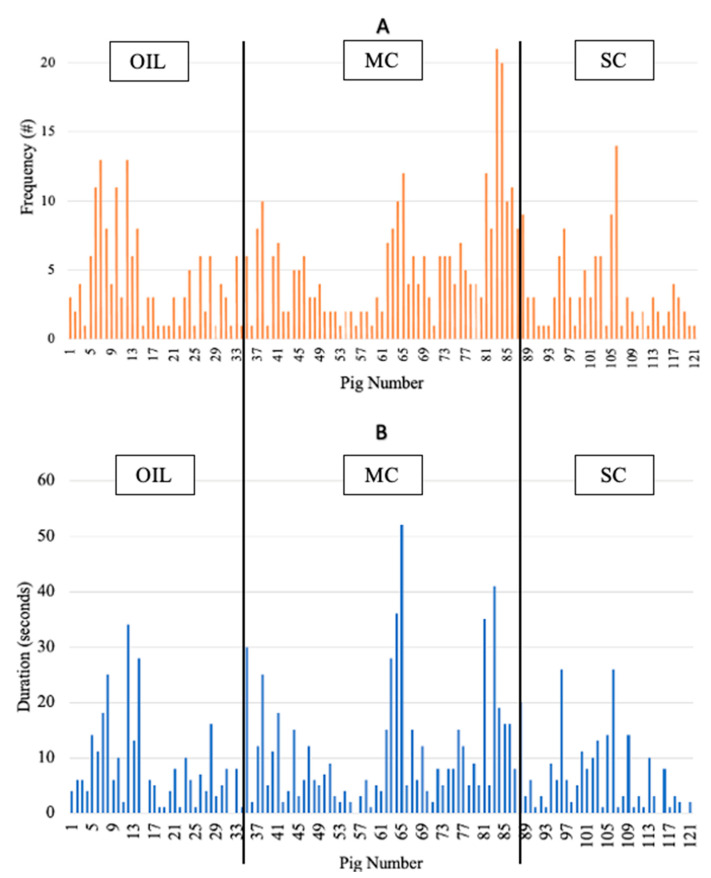
Descriptive measures of piglet individual variation in total frequency (**A**) and duration (**B**) of piglet purposeful investigations with environmental enrichment ropes on Day 2, the first day of placement. OIL = sunflower oil, MC = milky cheese, and SC = semiochemical.

**Figure 5:**
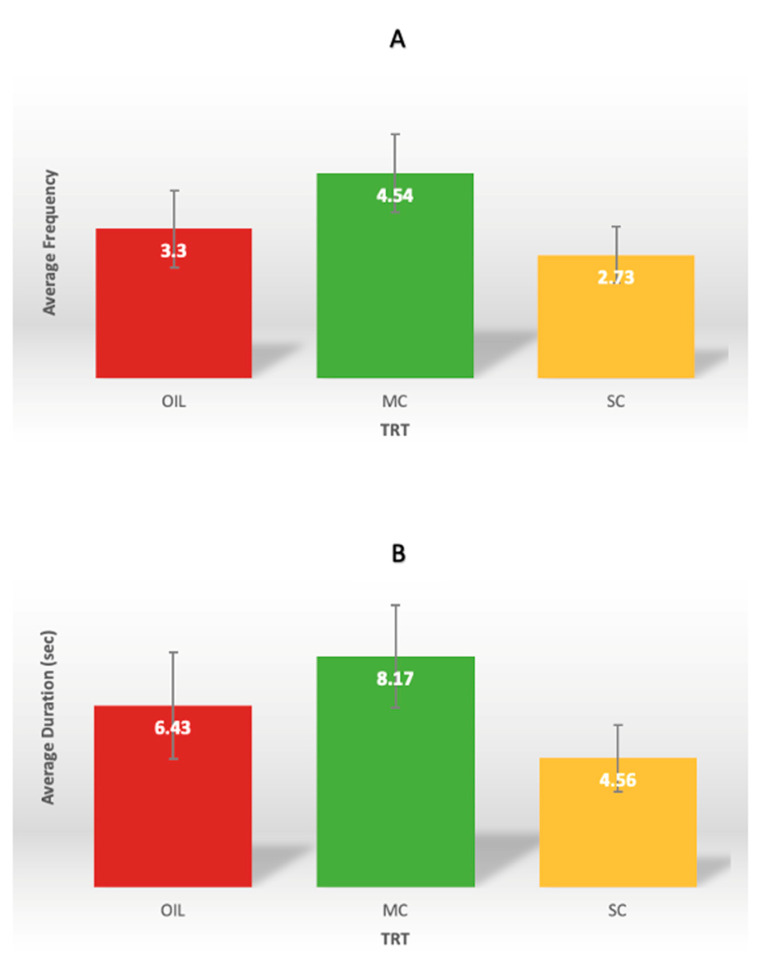
Average (±SE) frequency (**A**) and duration in seconds (**B**) of piglet purposeful investigations with environmental enrichment ropes on the first day of placement (Day 2 relative to farrowing). Ropes were dipped in one of three treatments: OIL = sunflower oil, MC = milky cheese, and SC = semiochemical. Frequency (Figure 5A; *p* = 0.20) and duration (Figure 5B; *p* = 0.21) of interactions was not different between treatments.

Average frequency and duration of investigations across all treatments were analyzed descriptively and will be presented as the means ± SD. Ropes were exposed to 161 piglets, of which 121 piglets (75%) interacted with ropes at least once. Of the piglets that interacted with the rope, there was a high level of individual variation in investigational total frequency (1 to 21) and duration (1 to 52 s) (Figure 4). Regardless of rope treatment, piglets displayed on average 4.5 ± 3.81 total frequency of investigations, and the average total time spent interacting with the ropes was 9 ± 9.4 s, respectively. On average, a single bout lasted 1 s (range < 1–14). Environmental enrichment rope treatment was not a source of variation for frequency (Figure 5A; *p* = 0.20) or duration (Figure 5B; *p* = 0.21) of investigations.

### 3.2. Rope Characteristics

**Figure 6:**
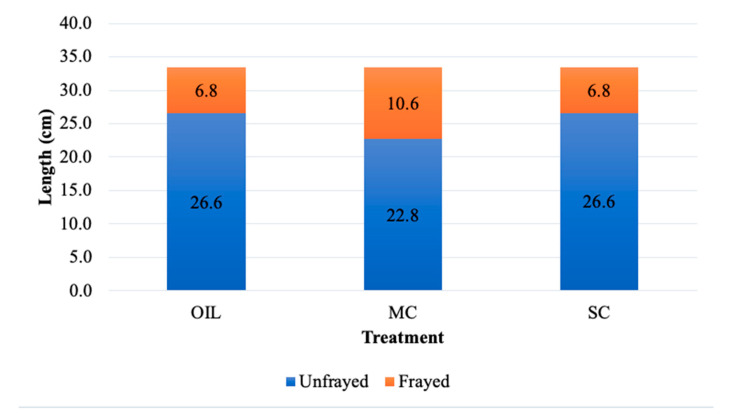
Descriptive measures of environmental enrichment rope length after removal from the farrowing stall 24 h after placement. The total rope length consisted of frayed and unfrayed sections. Ropes were dipped in one of three treatments: OIL = sunflower oil, MC = milky cheese, and SC = semiochemical.

Rope characteristics data are presented descriptively as the means ± SD. All placed ropes measured 33.0 cm in length with 2.5 cm of fraying at the rope end. At removal, mean rope length regardless of treatment was 33.4 ± 0.8 cm and mean rope fray length was 8.4 ± 6.6 cm. By month, the average rope length at removal was 34 ± 0.1 cm in July and 33 ± 0.1 cm in August, and rope fray length was 13.8 ± 5.2 cm in July and 2.8 ± 0.6 cm in August. Average rope length and rope fray length by treatment can be found in Figure 6. 

### 3.3. Mortality and Weight Gain

Environmental enrichment rope treatment was not a source of variation for litter average weight gain (OIL 9.5 ± 1.01 kg, SC 9.2 ± 0.98 kg, MC 8.4 ± 0.84 kg; *p* = 0.71). A total of 79 piglets died in the preweaning period, for a total preweaning mortality of 38% across all treatments. Of this number, 41 piglets died before the enrichment ropes were added (Days 0 to 1), 16 piglets died while the enrichment ropes were in the stalls (Days 2 to 5), and 22 piglets died after the enrichment ropes were removed (Days 6 to weaning). The 16 piglets that died from Days 2 to 5 were necropsied. Half (8 of 16) of these piglets were identified as dead by crushing, and 3 piglets were identified with no milk in their stomach (Table 1). Of the 16 piglets that died during the experimental period (Days 2 to 5), 68.8% (11/16) did not touch an enrichment rope on Day 2. Four of those piglets died on Day 2, and none of those piglets touched an enrichment rope on Day 2. 12 of the piglets died from Days 3 to 5, and only 5 (41.7%) of those piglets had touched an enrichment rope on Day 2. 

Piglets in the MC had the lowest percent mortality while the enrichment was in the stall (*p* = 0.01), piglets in SC had the lowest percent mortality after the enrichment was removed (*p* < 0.0001), and piglets in SC had the lowest percent mortality over the entire preweaning period affected by the rope enrichment (Days 2 to weaning; *p* = 0.03; Table 2). 

## 4. Discussion

This proof-of-concept study determined if neonatal piglets would interact with an environmental enrichment rope on Day 2, the first day of placement, and if this interaction would reduce preweaning mortality. There were three specific objectives to achieve this aim: (1) to describe piglet enrichment use; (2) to evaluate the impact of three attractants on piglet enrichment use; (3) to evaluate the enrichment impact on piglet growth rate and livability.

There is potential for a well-designed enrichment to entice neonatal piglets away from their sow after suckling to decrease chance of crushing. The environmental enrichment device used in this study was designed around neonatal piglet behaviors and anatomy. Neonatal piglets are cyclic in their behavior, with suckling, resting and heat seeking behaviors dominating [4]. Previous pig enrichment work has concluded that they are oral-nasal-facial-orientated [13]. Weaned and growing pigs are attracted to EE that is odorous, deformable, destructible, and chewable material [11], and hung at eye level [12]. While caution needs to be taken when extrapolating older pig behavior and enrichment to the neonate, previous work can serve as a useful starting point. The neonatal piglet is attracted to milk [26] and has a high sucking reflex [27]. Furthermore, neonate piglets are precocial, which means they can move and navigate their environments shortly after birth [28]. 

Each farrowing stall had a solid-wall partition that would not allow enrichment ropes to be tied directly to it. Thus, the enrichment device was designed to allow for each rope to hang at the optimal placement position and height within the farrowing stall. The device was cost effective, easy to build and place, and it did not require additional hardware to be secured. Biosecurity was considered, and although in this study biological samples were not taken before, during, and after cleaning, PVC and eyebolts could easily be removed and cleaned between farrowing groups. In total, 10 enrichment devices were built for this experiment, and the total cost of all the enrichment device materials (PVC, eyebolts, washers, nuts, carabiner hooks, and rope) was $330, or $33 per device. Note, this cost does not include the price of the enrichment treatments or labor.

The enrichment ropes were placed into the farrowing stalls Day 2 after farrowing, and not immediately after birth. This timing was justified for two reasons. First, it allowed farm staff to perform normal post-farrowing processing and piglet management procedures. Second, it prevented any sow-piglet interference that may have detrimentally affected colostrum intake and/or teat order establishment. As there was no treatment impact on litter weight gain in this study, there would be value in future work adding this device before piglets have arrived. 

Physical enrichment rope characteristics before and after placement were collected to provide an indirect measure of piglet enrichment use. No damage or visible missing parts of the rope were noted at removal, which is consistent with observations when ropes have been used to collected oral fluids [29]. Many ropes were visibly dirty upon removal, indicating piglets contacted the ropes. Ropes and fray length were longer at removal than at placement. This may be explained by the rope unwinding over time due to piglet contact. The increase in rope length and fray was similar across treatments. Casual observation suggests that average rope length and fray was longer in July than in August and may be partially explained by a change in rope spool between these months. 

Overall, the high level of interaction by piglets in the Day 2 environment after weaning is an important and novel finding, as it suggests that even at this young age, piglets are attentive to and interacting with their environments. Piglet interest in the ropes on Day 2 suggests that environmental enrichments are practical for piglets even at this young age. 

To achieve a comparison between levels of investigations across treatment groups, individual piglet investigations were summed by treatment and then analyzed. Therefore, the lack of differences between frequency and duration of investigations by treatment may be at least partly explained by the high individual variation of EE use by individual piglets that was not accounted for in the analysis of the experimental unit, the litter. It is also important to note that while there were no behavioral differences between treatments, numerically the MC treatment group had higher frequency and duration of investigations. Previous studies that used MC flavorings support that it is not aversive to piglets in creep feed or as flavor cues and was preferred by piglets under prenatal flavor exposure conditions [16,17]. It was interesting that in this study, neonatal piglets did not interact more or for longer with the SC. Aviles-Rosa et al. [15] noted a preference for feeders sprayed with SC in nursery pigs. 

While piglets were observed interacting with the EE, an important unanswered question is how often and how long does a piglet need to interact with the enrichment to receive benefits in terms of livability, growth, and/or welfare? The high individual variation both within and between litters could provide a starting point, and future studies investigating the relationship between individual piglet enrichment use and performance measures are warranted. In this study, although there was not a significant difference across treatments in piglet rope interactions, there was an effect of rope treatment on piglet mortality. It would be recommended that future work measure piglet distance from and time spent near the enrichment ropes, because some piglets may not directly interact with the ropes but still may be drawn away from the sow, thus reducing the risk of crushing. 

There are concerns that placement of EE could interfere with piglet nursing behavior; however, of the 75% of piglets interacting with the ropes on Day 2, none died. This is an interesting finding that may warrant further investigation. Of the 16 piglets that died during the study period, half were attributed to crushing, and only three did not have milk present in their stomachs. Although half of the piglets that died were assigned to the MC treatment, this treatment group had more sows and larger litters. 

Our results suggest that there is potential that the enrichment treatments can impact piglet livability, and we suggest that future work needs to take this enrichment concept into a larger commercial environment to determine if neonatal welfare is improved during lactation. 

## 5. Conclusions

When provided with environmental enrichment designed with neonatal piglet anatomy and behavior considerations, piglets showed high individual variation in their investigations of enrichment. A total of 75% of piglets touched the ropes at least once on Day 2, indicating that neonate piglets are attentive to their environment early in life. There was a high variation in frequency and duration of individual piglet investigations across all treatments. The milky cheese enrichment was investigated the most by piglets and had the lowest mortality during the enrichment period (Days 2 to 5). This proof-of-concept study provides pivotal information for implementing environmental enrichment into larger commercial scale studies. 

## Figures and Tables

**Table 1:** Descriptive results of number (#) of piglets that died on Day 2 to 5, when the enrichment ropes were present in the farrowing stall. All piglets were necropsied to identify those that were crushed ^1^ and to check for milk presence in the stomach ^2^.

	Treatments ^3^			Total
	OIL	SC	MC	
# of sows	7	8	11	26
# of piglets on Day 2	41	51	69	161
Crushed + milk	1	1	6	8
Crushed + no milk	0	0	0	0
Not crushed + milk	3	1	1	5
Not crushed + no milk	0	2	1	3
Total # piglets dead	4	4	8	16

^1^ Piglets were classified as crushed through external signs of trauma, including bruising (injury that included discoloration and inflammation of the skin without exposure of underlying tissues) and compression of parts of the body, or if they were found underneath the sow; ^2^ piglets stomach contents were checked and classified as milk present (milk) or milk not present (no milk); ^3^ ropes dipped in OIL = sunflower oil; SC = semiochemical; MC = milky cheese.

**Table 2:** Preweaning performance measures of litters provided with environmental enrichment ropes over Days 2 to 5 after farrowing. Results are presented as the LSMeans ± SE where statistical analysis was performed, or as the mean ± SD where results are presented descriptively.

	Treatments ^1^			*p*-Value
	OIL	SC	MC	
Number of sows	7	8	11	
Average sow parity	4	3.8	4	
Average litter size (#)	6.3 ± 3.09	8.0 ± 4.00	8.6 ± 3.21	
Average weaning age (d)	20.3 ± 1.89	20.1 ± 1.96	19.6 ± 2.66	
	**Litter Body Weight (kg)**			
Average birth weight	3.0 ± 0.48	3.0 ± 0.70	3.3 ± 0.38	
Average weaning weight	12.4 ± 2.89	12.3 ± 2.20	11.9 ± 2.94	
Average weight gain	9.5 ± 1.01	9.2 ± 0.98	8.4 ± 0.84	0.71
	**Litter Mortality (%) ^2^**			
Days 2 to 5 ^3^	10.7 ± 1.29 ^a^	9.2 ± 1.07 ^a^	6.1 ± 0.74 ^b^	0.01
Days 6 to weaning ^4^	7.4 ± 1.03 ^a^	4.0 ± 0.65 ^b^	10.8 ± 0.98 ^c^	<0.0001
Days 2 to weaning ^5^	19.2 ± 1.71 ^a^	13.7 ± 1.29 ^b^	18.2 ± 1.27 ^a^	0.03

^1^ Ropes dipped in OIL = sunflower oil; SC = semiochemical; MC = milky cheese; ^2^ average percent mortality (%), calculated as the average number of piglets dead at the end of the time period / the average number of piglets alive at the beginning of the time period × 100; ^3^ Days 2 to 5, when enrichment ropes were in the stall; ^4^ Days 6 to weaning, after enrichment ropes were removed from the stall. Weaning age ranged from 15 to 22 days of age; ^5^ Days 2 to weaning mortality, defined as all piglets that were alive at rope placement and dead before weaning; ^a,b,c^ Different superscripts within a row indicate significance at *p* ≤ 0.05.

## Data Availability

The data presented in this study are available on request from the corresponding author. The data are not publicly available due to further data analysis to be conducted and published by the research team.
